# Supplementary material for: Epithelial-to-Mesenchymal Transition and Phenotypic Marker Evaluation in Human, Canine, and Feline Mammary Gland Tumors
Source: Animals (Basel). 2023 Feb 28;13(5):878. doi: 10.3390/ani13050878 (PMC10000046; doi:10.3390/ani13050878)
Supplement: Supplementary file 1 [file animals-13-00878-s001.zip › animals-2210231-supplementary.pdf]

Table S1. Immunohistochemical expression of estrogen (ER) and progesterone (PR) receptors and ERBB2 in the samples.

Species	Sample ID	ER (%)	PR (%)	ERBB2
HBC ER+	1	90	15	2+
HBC ER+	2	90	90	1+
HBC ER+	3	70	90	0
HBC ER+	4	100	100	1+
HBC ER+	5	90	50	1+
HBC ER+	6	50	0	0
HBC ER+	7	80	40	0
HBC ER+	8	90	70	1+
HBC ER+	9	90	90	1+
HBC ER+	10	70	80	0
HBC TNBC	11	0	0	0
HBC TNBC	12	0	0	1+
HBC TNBC	13	0	0	1+
HBC TNBC	14	0	0	1+
HBC TNBC	15	0	0	0
HBC TNBC	16	0	0	0
HBC TNBC	17	0	0	0
HBC TNBC	18	0	0	0
HBC TNBC	19	0	0	0
HBC TNBC	20	0	0	0
HBC TNBC	21	0	0	0
CMT grade I	1	53	NP	0
CMT grade I	2	21	NP	0
CMT grade I	3	13	NP	0
CMT grade I	4	15	NP	0
CMT grade I	5	62	NP	0
CMT grade I	6	27	NP	0
CMT grade I	7	37	NP	0
CMT grade I	8	27	NP	0
CMT grade I	9	49	NP	0
CMT grade I	10	15	NP	0
CMT grade I	11	14	NP	0
CMT grade II	12	2	NP	0
CMT grade II	13	2	NP	0
CMT grade II	14	7	NP	0
CMT grade II	15	57	NP	0
CMT grade II	16	3	NP	0
CMT grade II	17	13	NP	0
CMT grade II	18	9	NP	0
CMT grade II	19	21	NP	0
CMT grade II	20	23	NP	0
CMT grade II	21	2	NP	0
CMT grade II	22	5	NP	0
FMT	1	0	0	0
FMT	2	0	0	0
FMT	3	0	0	0
FMT	4	0	0	0

FMT	5	0	0	0
FMT	6	0	0	0
FMT	7	0	0	0
FMT	8	0	0	0
FMT	9	0	0	0
FMT	10	0	0	0
FMT	11	0	0	0
FMT	12	0	0	0

HBC, human breast cancer; CMT, canine mammary tumor; FMT, feline mammary tumor
